# The Related Factors and Effect of Electrode Displacement on Motor Outcome of Subthalamic Nuclei Deep Brain Stimulation in Parkinson’s Disease

**DOI:** 10.3390/jcm12247561

**Published:** 2023-12-08

**Authors:** Tianshuo Yuan, Yingchuan Chen, Guanyu Zhu, Jianguo Zhang

**Affiliations:** 1 Department of Neurosurgery, Beijing Tiantan Hospital, Capital Medical University, Beijing 100070, China; 2 Department of Functional Neurosurgery, Beijing Neurosurgical Institute, Capital Medical University, Beijing 100070, China; 3 Beijing Key Laboratory of Neurostimulation, Beijing 100070, China

**Keywords:** brain shift, brain atrophy, deep brain stimulation, electrode displacement, pneumocephalus, Parkinson’s disease

## Abstract

Background: Previous studies have revealed the existence of electrode displacement during subthalamic nucleus deep brain stimulation (STN-DBS). However, the effect of electrode displacement on treatment outcomes is still unclear. In this study, we aimed to analyze the related factors of electrode displacement and assess postoperative electrode displacement in relation to the motor outcomes of STN-DBS. Methods: A total of 88 patients aged 62.73 ± 6.35 years (55 males and 33 females) with Parkinson’s disease undergoing STN-DBS, with comprehensive clinical characterization before and 1 month after surgery, were involved retrospectively and divided into a cross-incision group and cannula puncture group according to different dura opening methods. The electrode displacement, unilateral pneumocephalus volume percent (uPVP), and brain volume percent were estimated. Results: A significant anterior and lateral electrode displacement was observed among all implanted electrodes after pneumocephalus absorption (*p* < 0.0001). The degree of electrode displacement was positively correlated with the uPVP (*p* = 0.005) and smaller in females than males (*p* = 0.0384). Electrode displacement was negatively correlated with motor improvement following STN-DBS in both on-medication and off-medication conditions (*p* < 0.05). Dural puncture reduced the uPVP (*p* < 0.0001) and postoperative electrode displacement (*p* = 0.0086) compared with dural incision. Conclusions: Electrode displacement had a negative impact on the therapeutic efficacy of STN-DBS. Opening the dura via cannula puncture is recommended to increase the accuracy of the lead implantation.

## 1. Introduction

Subthalamic nucleus deep brain stimulation (STN-DBS) is the most effective surgical treatment for patients with advanced Parkinson’s disease (PD) [1]. It can relieve both motor symptoms, such as tremor, rigidity, bradykinesia, and postural abnormalities [2,3], and non-motor symptoms, including anxiety, depression, and pain [4,5]. The precise implantation of electrodes into the sensorimotor area of the STN is considered paramount for maximizing the clinical effectiveness [6,7]. The location of depth electrodes with respect to the surrounding brain tissue has a large impact on the volume of tissue activated (VTA) and the activation of corresponding circuits [8,9]. However, numerous factors limit the surgical accuracy of DBS, including air inflow and cerebrospinal fluid loss during stereotactic procedures, which may influence the efficacy of STN-DBS [10,11]. Pneumocephalus begins to occur as soon as the skull and dura are opened and usually resolves over the weeks following operation, which might cause an intraoperative brain shift and postoperative electrode displacement [12,13].

Previous studies have reported a correlation between the volume of pneumocephalus and degree of DBS electrode displacement [10,14]. Such electrode displacement might influence the choice of active contact and parameter settings during the first months of follow-up [11]. Although no relationship has been found between brain shifts and motor improvement following STN-DBS [15], the correlation between electrode displacement and the efficacy of STN-DBS remains under investigation. Furthermore, Horn et al. [16] suggested that brain shift correction should be applied to avoid a registration error during the analysis of immediate postoperative imaging, which was not performed in the previous studies [10,14,17]. This inaccurate registration might lead to an incorrect conclusion due to the influence of a brain shift.

**Figure 1:**
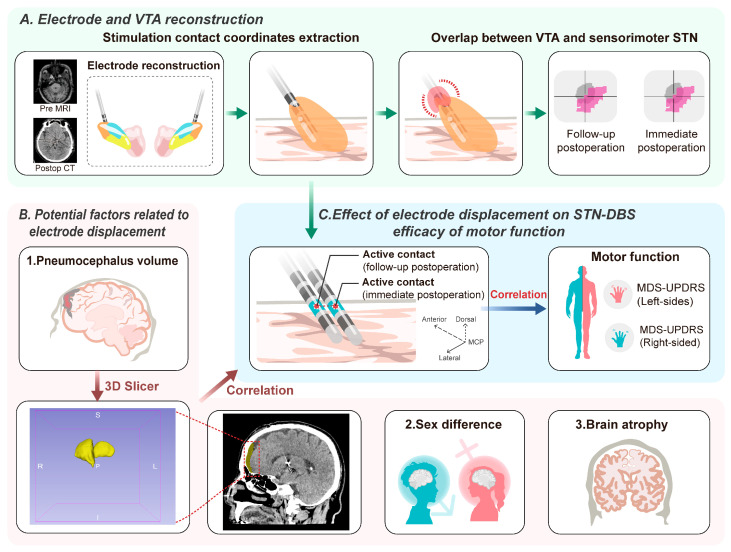
Image processing and correlation analysis workflow. (**A**) The overall procedure of electrode and VTA reconstruction. Extracting active contact coordinates to calculate the displacement and obtain the overlapping volume to reflect the relative position between the active contact and sensorimotor area of the STN. (**B**) Analyzing the correlation between potential related factors and electrode displacement. (**C**) Investigating the effect of electrode displacement on the motor improvement of the contralateral body side. STN-DBS: subthalamic nucleus deep brain stimulation; VTA: volume of tissue activated; MDS-UPDRS: Movement Disorder Society Unified Parkinson’s Disease Rating Scale.

In the current study, we investigated whether electrode displacements did exist with the consideration of brain shift interference, analyzed the effect of electrode displacement on the outcomes of STN-DBS (Figure 1), and provided a feasible method to reduce the impact of pneumocephalus on electrode displacements.

## 2. Materials and Methods

### 2.1. Participants

A total of 88 patients (33 females) with PD treated with bilateral STN-DBS at Beijing Tiantan Hospital between October 2018 and June 2021 were retrospectively studied. These patients were divided into two groups according to different dura opening methods during two successive periods of time. This study was conducted following approval by the Ethics Committee of Beijing Tiantan Hospital (KY 2022-006-02), and all protocols were performed in accordance with the Declaration of Helsinki. All patients provided written informed consent. Details for inclusion and exclusion criteria are described in the supplemental methods (Supplementary Figure S1).

### 2.2. STN-DBS Implantation and Image Acquisition

**Figure 2:**
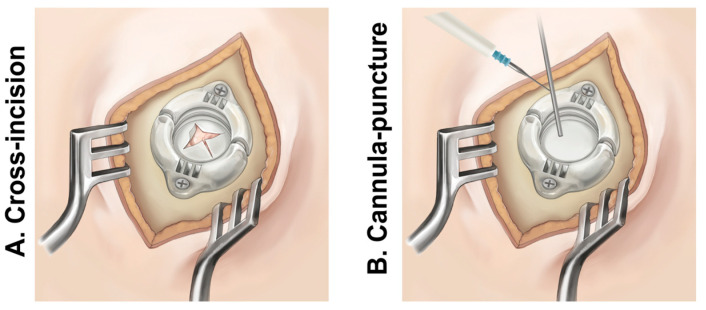
Two methods of opening the dura. (**A**) Opening the dura via cross-incision. (**B**) Opening the dura via cannula puncture with the assisted of monopolar coagulation.

Lead implantation was performed using a Leksell microstereotactic system and assisted by a Leksell Surgiplan workstation (Elekta Instrument AB, Stockholm, Sweden). Preoperative frame-based computer tomography (CT) and preoperative magnetic resonance imaging (MRI) data were imported and fused in the planning system to determine the target and trajectory. All patients were placed in the semi-supine position, and DBS electrodes were implanted under local anesthesia. The location of the burr hole was determined according to the cortical entry point of the planned trajectory. The dura was opened using one of the following two methods: cross-incision or puncture with a steel cannula with the assistance of monopolar coagulation (Figure 2). After, the dural defect was sealed with fibrin glue. Intraoperative microelectrode recording (MER) was performed with one microelectrode in each hemisphere by the NeuroOmega system (Alpha Omega Engineering, Israel) to assess the optimal electrode location. Electrophysiological signals were recorded to reveal entry into and exit from the STN to guide the implantation of the DBS electrodes. After a standard STN signal was identified, the quadripolar DBS electrode (model 3389, Medtronic, Minneapolis, MN, USA, or model L301, Pins Medical, Beijing, China) was implanted and connected to a temporary stimulator to assess the stimulation effectiveness and side effects. If the effects of the temporary stimulation were satisfactory, the electrodes would be fixed at the burr hole with lead anchoring devices and connected to an implantable pulse generator for permanent electrode implantation.

Presurgical imaging was performed on a 3.0T MRI system (Ingenia, Philips Medical Systems, Best, The Netherlands), and three-dimensional (3D) T1 images were acquired from each participant. Postoperative CT (thickness: 0.625 mm) was performed immediately (4 to 10 h) and 1 month (4 to 5 weeks, when no pneumocephalus was detected) after STN-DBS (Supplementary Figure S2). Details of the scan parameters are shown in the Supplemental Methods.

### 2.3. Clinical Evaluation and Postoperative Programming

Motor function was assessed using Movement Disorder Society Unified Parkinson’s Disease Rating Scale III (MDS-UPDRS III) at baseline and 1 month after surgery [18]. Left-sided and right-sided motor scores (MDS-UPDRS items 3.3b–e, 3.4–3.8, 3.15, 3.16, and 3.17a–d) were separately analyzed. It should be noted that axial motor scores were not included in the current study. Clinical evaluations were conducted during the preoperative visit and 1 month (approximately 4–5 weeks) post-surgery. We performed an off-medication assessment after a minimum 12 h withdrawal period from dopaminergic medications, whereas on-medication assessments were performed after the patients took medication at the evaluation center. The patients returned to the hospital for pulse generator activation at 1 month post-operation. The effectiveness and safety of each electrode contact were assessed with monopolar stimulation by trained neurosurgeons, and they were observed for 3–7 days to establish a stable STN-DBS setting. Subsequently, postoperative assessments were conducted under the on-stimulation condition. The levodopa equivalent daily dose (LEDD) [19] and stimulation settings were recorded after each evaluation.

### 2.4. Electrode Localization and Estimation of the Stimulation Volume

The localization of the DBS electrode contacts and the calculation of corresponding VTAs were implemented using Lead-DBS software 2.5.2 [16]. DBS electrodes were localized after brain shift correction using the automatic PaCER algorithm [20], which requires very little manual refinement compared to other methods. After automated reconstruction, two researchers checked the accuracy, and manual adjustment was conducted for inaccurate electrodes. Then, the stereotactic X-(lateral), Y-(anteroposterior), and Z-(dorsoventral) distances of the center of the contact relative to the midcommissural point were extracted with MATLAB 2021a (Mathworks, Natick, MA, USA). The coordinates of the active contact were collected based on the individual programming settings, and all left coordinates were reversed to the right side for further processing. The Euclidean distance (ED) from the position of the active contact immediately after surgery to the position at 1-month follow-up was calculated for each individual’s contralateral body side as follows: $\sqrt{{\left(\Delta X\right)}^{2}+{\left(\Delta Y\right)}^{2}+{\left(\Delta Z\right)}^{2}}$.

The VTA was estimated in native space based on the individual stimulation parameters using a finite element method approach established with the SimBio-FieldTrip pipeline [21]. The volume of the sensorimotor STN in the DISTAL Minimal Atlas [22] was transferred to native space using a reverse deformation field in each patient. To evaluate the difference in the relative distance between the active contact and neuromodulatory target during pneumocephalus absorption, we analyzed the overlap between the VTA and sensorimotor area of the STN in both the immediate and follow-up postoperative imaging studies. Details are shown in the Supplement Methods.

### 2.5. Individual Brain Atrophy and Pneumocephalus Volume Measurement

We used FreeSurfer 6.0 to automatically segment the total intracranial volume (ICV) and total brain volume [23]. Brain parcellation and segmentation were conducted using the standard “recon-all” script, and quality control was applied with ENIGMA Cortical Quality Control Protocol 2.0 (http://enigma.ini.usc.edu/, accessed on 18 June 2023). The pneumocephalus volume of each hemisphere was measured using immediate postoperative CT images via a semi-automatic method based on 3D slicer software 5.1.0 (https://www.slicer.org, accessed on 18 June 2023) [24], and slice-by-slice quality control was subsequently conducted by two neurosurgeons (Figure 1). The ICV was used to correct for interindividual differences in total brain size [25]. Brain atrophy was defined as the total brain volume as a percentage of the ICV. The unilateral pneumocephalus volume percent (uPVP) was calculated as the ratio of unilateral pneumocephalus volume to the ICV.

### 2.6. Statistical Analysis

The data are presented as means ± standard deviations (SD) or medians (25th and 75th percentiles), and parametric (paired samples *t*-test or independent samples *t*-test) and nonparametric (paired Wilcoxon rank sum test or Mann–Whitney U tests) statistics were used to evaluate potential differences. The categorical variables, expressed as numbers (%), were compared using a chi-squared test. We used generalized linear models (GLMs) to identify factors related to the ED of the contact displacement and the efficacy of STN-DBS, and then we performed a false discovery rate (FDR) correction [26], adjusting the *p*-values for GLMs. The variance inflation factor (VIF) was calculated for each variable to avoid multicollinearity [27]. A VIF < 5 was considered acceptable.

The levodopa response and motor improvement after STN-DBS in the off-medication condition and in the on-medication condition were calculated as follows:

$$Levodopa\;response={(MDS\text{-}UPDRS}_{pre:med\text{-}off}-{MDS\text{-}UPDRS}_{pre:med\text{-}on})/{MDS\text{-}UPDRS}_{pre:med\text{-}off}\times 100\%,\;Motor\;improvement\;OFF=({MDS\text{-}UPDRS}_{pre:med\text{-}off}-{MDS\text{-}UPDRS}_{post:stim\text{-}on\&med\text{-}off})/{MDS\text{-}UPDRS}_{pre:med\text{-}off}\times 100\%,\;Motor\;improvement\;ON=({MDS\text{-}UPDRS}_{pre:med\text{-}on}-{MDS\text{-}UPDRS}_{post:stim\text{-}on\&med\text{-}on})/{MDS\text{-}UPDRS}_{pre:med\text{-}on}\times 100\%.$$

If the scale scores had 0 as the denominator, score changes were calculated instead of the percentage of improvement for comparison. Statistical analyses were conducted using MATLAB 2021a (Mathworks, Natick, MA, USA) and SPSS 27 (IBM, Chicago, IL, USA). *p* < 0.05 was considered significant for all tests.

## 3. Results

### 3.1. Patient Characteristics

The study sample comprised 88 PD patients (33 females) with 176 STN leads, who were divided into two groups according to the dura opening method, including 53 patients (ages 52 to 78 years) in the cross-incision group and 35 patients (ages 52 to 76 years) in the cannula puncture group. No difference was found in the baseline characteristics between the two groups (Table 1).

### 3.2. Electrode Displacement and Related Factors

A total of 176 electrodes were analyzed to determine the orientation of the electrode displacement. Compared with the active contact position in the immediate postoperative period, the displacement was significantly more lateral (0.29 ± 0.44 mm, *p* < 0.0001) in the stereotactic X-axis and more anterior (0.62 ± 0.39 mm, *p* < 0.0001) in the stereotactic Y-axis, whereas the displacement in the stereotactic Z-axis was not significant (*p* = 0.31). The ED of the active contact displacement was 0.93 (0.76, 1.20) mm, indicating a considerable electrode displacement.

**Figure 3:**
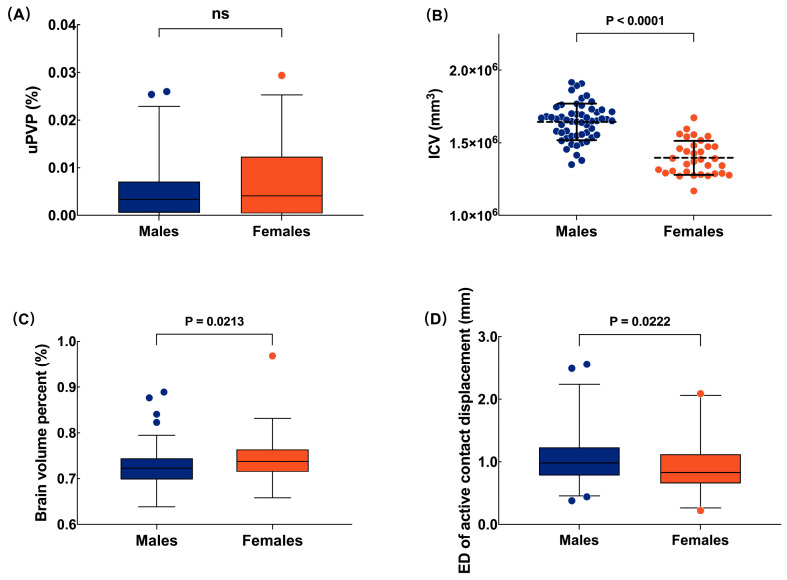
Sex differences in preoperative brain structure and postoperative electrode displacement. Data with non-normal distribution are presented as box plots (median (25th and 75th percentiles)). Data with normal distribution are presented as scatter plots (mean ± standard deviation). (**A**) No difference was found in the uPVP between sexes. (**B**) The ICV of males was larger than that of females. (**C**) Males had more severe brain atrophy than females. (**D**) Males had a greater ED of contact displacement than females. uPVP: unilateral pneumocephalus volume percent; ICV: intracranial volume; ED: Euclidean distance; ns: no significance.

The GLM showed that the uPVP was significantly and positively correlated with the ED of the active contact displacement (corrected *p* = 0.005). The ED of the active contact displacement was smaller in females than males (corrected *p* = 0.0384). No correlation was found between brain atrophy and electrode displacement (Table 2). Although there was no significant difference in the uPVP between the different hemispheres (*p* = 0.5669) and different sexes (*p* = 0.2485, Figure 3A), the results of the independent t-test suggested that the males had a significantly larger ICV (*p* < 0.0001, Figure 3B), more severe brain atrophy (*p* = 0.0213, Figure 3C), and a greater ED of active contact displacement (*p* = 0.0222, Figure 3D) than the females. More details are shown in Supplementary Table S1.

### 3.3. The Effect of Electrode Displacement on Clinical Outcomes

The motor improvement associated with STN-DBS was calculated to obtain a correlation analysis based on GLMs (Table 3). We found the ED of the active contact displacement was significantly and negatively correlated with the motor efficacy of the STN-DBS on the contralateral body side under both the on-medication (corrected *p* = 0.0313) and off-medication conditions (corrected *p* = 0.0209). The brain volume percent was significantly and positively correlated with an improvement in motor symptoms under the on-medication condition (corrected *p* = 0.0313). Males exhibited a significantly greater improvement in motor function following STN-DBS than females under the on-medication condition (corrected *p* = 0.0031). The baseline levodopa response was significantly and negatively correlated with an improvement in motor symptoms under the on-medication condition (corrected *p* < 0.0001). The overlap between the VTA and sensorimotor area of the STN was significantly larger in the follow-up period (12.82 (5.82, 21.53) mm^3^) in comparison with that in the immediate postoperative period (11.33 (3.67, 20.19) mm^3^, *p* = 0.001). 

### 3.4. Modification of the Dural Opening Technique

**Figure 4:**
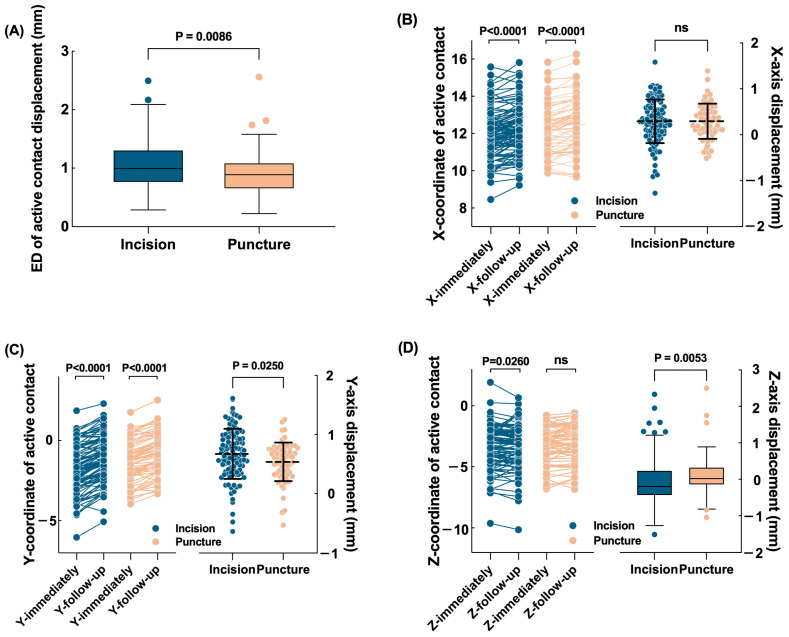
Differences in electrode displacement between the cross-incision group and cannula puncture group. Data with non-normal distribution are represented with box plots (median (25th and 75th percentiles)). Data with normal distribution are represented with scatter plots (mean ± standard deviation). (**A**) The ED of active contact displacement was greater in the cross-incision group than that in the cannula puncture group. (**B**) The X-coordinate of the active contact was more lateral in the follow-up period in both groups. No difference in X-axis displacement was found between the two groups. (**C**) The Y-coordinate of the active contact was more anterior in the follow-up period in both groups, but the Y-axis displacement was greater in the cross-incision group. (**D**) Z-axis displacement was found in the cross-incision group but not in the cannula puncture group. ED: Euclidean distance; ns: no significance.

In the final part, we compared the effects of different dura opening methods on pneumocephalus and electrode displacement. The uPVP in the cross-incision group was significantly larger than that in the cannula puncture group (*p* < 0.0001). The cross-incision group had a longer ED of active contact displacement compared with the cannula puncture group (*p* = 0.0086, Figure 4A). In the X-axis and Y-axis directions, lateral (*p* < 0.0001, Figure 4B) and anterior (*p* < 0.0001, Figure 4C) displacements were found in both groups, but the cross-incision group had a greater degree of Y-axis displacement than the cannula puncture group (*p* = 0.025, Figure 4C). In the Z-axis direction, a ventral displacement was found in the cross-incision group (*p* = 0.026), whereas no Z-axis displacement was found in the cannula puncture group (Figure 4D). There was no difference in motor improvement between the two groups under either the on-medication (*p* = 0.6015) or off-medication conditions (*p* = 0.9486).

## 4. Discussion

The aim of this study was to better understand the relationship between electrode displacement and the efficacy of STN-DBS on motor function. In this study, we confirmed the occurrence of lead displacement in STN-DBS, investigated the factors related to electrode displacement, and inferred that the greater the electrode displacement distance, the less improvement there was in motor function.

### 4.1. Postoperative Electrode Displacement and Related Factors

Previous studies have demonstrated that DBS electrodes may not reach their final positions upon immediate postoperative imaging. van den Munckhof et al. [10] enrolled 14 PD patients who underwent STN-DBS and analyzed the stereotactic coordinates of the metallic artifact of the deepest contact on the immediate postoperative and follow-up CT scans. They found that the electrode position in the immediate postoperative period was displaced medially, posteriorly, and ventrally in comparison with that in the follow-up period. Göransson et al. [28] found that the lead tip displayed a tendency to move laterally, anteriorly, and inferiorly. However, the results of previous studies regarding the orientation of electrode displacement have not been consistent, and this may result from inaccurate registration due to neglecting the impact of brain shifts. Indeed, brain shifts of up to 4.0–5.6 mm have been observed in deep brain structures, including the AC, PC, and putamen [29,30]. If the brain shift effect is not taken into account, to some degree, the electrode displacement is more likely to refer to the skull instead of the modulated target. In recent times, artificial intelligence and automated image analyses have been utilized to establish predictive models and identify potential mechanisms [21,31] that contribute to enhancing the precision of electrode reconstruction and reducing the effects of brain shifts. In the current study, automated advanced normalization tools [32] were used for image registration, and brain shift correction [33] was applied to reduce the registration error. Compared with the immediate postoperative period, the electrode displacement was observed in the anterior–lateral direction in the follow-up period. The degree of the electrode displacement was influenced by pneumocephalus. We also found that males had a greater distance of electrode displacement than females, although there was no difference in the uPVP between sexes. Thus, males who have a larger ICV than females might be more susceptible to pneumocephalus and experience a greater electrode displacement.

### 4.2. Impact of Electrode Displacement on STN-DBS Efficacy

We revealed that electrode displacement and brain atrophy had a significant relationship with postoperative motor outcomes. Decreased brain volume and increased electrode displacement were associated with reduced motor improvements following STN-DBS. The brain atrophy results were consistent with previous studies, which found that the ventricular volume and atrophy of the motor cortex and thalamus were associated with poor motor improvement after DBS [34,35]. In terms of electrode displacement, Choi et al. [36] analyzed the impact of brain shift on subcallosal cingulate DBS and found that electrode displacement decreased the connectivity between the electrode and ventral striatum. Petersen et al. [15] reported that there was no effect of brain shift on the clinical outcome of STN-DBS. Previous studies have investigated the effect of brain shift and electrode displacement separately; however, it is more meaningful to study the relative position change between the active contact and neuromodulation target. Therefore, a further analysis was conducted to estimate the overlap between the VTA and sensorimotor area of the STN in native space, which is considered to be positively correlated with the efficacy of STN-DBS on motor symptoms [37]. The result revealed that the overlap was significantly increased after pneumocephalus was resolved, indicating the relative position between the active contact and sensorimotor area of the STN in the follow-up period might be closer and more approximate to the preoperative planned condition after the brain returns to its preoperative position. 

### 4.3. Methods to Reduce the Impact of Electrode Displacement

The pneumocephalus volume is influenced by several factors, including the patient position, duration of surgery, burr hole localization, and the method of burr hole closure [11]. Here, we adopted a modification technique to reduce the pneumocephalus volume by opening the dura via puncture rather than incision. The results showed that both the pneumocephalus volume and the degree of electrode displacement, especially in the anterior and ventral direction, were significantly reduced in the cannula puncture group compared with the cross-incision group. However, no significant difference in motor improvement after STN-DBS was found between the two groups, which might be attributed to the insufficient statistical power resulting from the small sample size of patients included in the cannula puncture group. Meanwhile, we provided evidence that follow-up imaging was more accurate in reflecting the preoperative planning location than immediate postoperative imaging because of a larger overlapping volume between the VTA and sensorimotor area of the STN. Currently, many studies have proposed new programming methods (e.g., remote programming [38,39] and tract-based programming [40]), and these methods necessitate accurate high-resolution postoperative imaging. Therefore, long-term postoperative imaging is essential to confirm the electrode location and guide postoperative programming. Furthermore, well-established animal models with STN-DBS are utilized to explore therapeutic mechanisms and potential indications [41,42]. In the future, studies with animal models are needed to further investigate the mechanism of electrode displacement.

### 4.4. Limitations

Our analysis had several limitations that should be noted. First, it was conducted retrospectively based on previously archived data. Second, we included clinical outcomes at a near-term follow-up, and longer follow-up periods are needed to verify the long-term effect of electrode displacement, although other confounders could then be introduced, such as disease progression and medication adjustment. Moreover, MERs of all the hemispheres were conducted with a single microelectrode. The effect of a multichannel MER on pneumocephalus formation and electrode displacement still needs to be confirmed in future studies.

## 5. Conclusions

This study employed an automated image analysis to provide evidence for the negative impact of electrode displacement on the therapeutic efficacy of STN-DBS. The ED of the active contact displacement showed a negative correlation with motor improvement following STN-DBS. We therefore propose cannula puncture as a technique modification to reduce the impact of pneumocephalus and obtain optimal and less variable motor improvement for individual patients with PD undergoing STN-DBS.

## Figures and Tables

**Table 1:** Baseline characteristics and improvement in motor function.

	Cross-Incision	Cannula Puncture	*p*-Value
Age (year)	63.04 ± 6.22	62.26 ± 6.50	0.5775
Duration of disease (year) ^a^	8.00 (5.00, 12.00)	9.00 (7.00, 12.00)	0.3506
Sex (female, %) ^b^	21 (39.6%)	12 (34.3%)	0.6128
Hoehn–Yahr stage ^a^	3.00 (3.00, 3.00)	3.00 (3.00, 3.00)	0.2703
LEDD (mg/d) ^a^	718.25 (600.00, 837.75)	750.00 (600.00, 1127.38)	0.2455
Intracranial volume (mm^3^)	1,545,267.86 ± 185,510.70	1,559,875.98 ± 144,876.11	0.6984
Brain volume percent (%) ^a^	72.35% (70.35%, 73.80%)	73.53% (71.66%, 75.79%)	0.1034
uPVP (%) ^a^	0.50% (0.17%, 1.09%)	0.15% (0.01%, 0.55%)	**<0.0001**
MDS-UPDRS III ON ^a^	7.00 (4.00, 9.00)	5.00 (4.00, 7.75)	0.0747
MDS-UPDRS III OFF	15.74 ± 6.25	15.21 ± 6.45	0.5956
Levodopa response (%)	56.65% ± 20.97%	60.72% ± 16.49%	0.1749
Motor improvement ON	2.30 ± 4.66	2.67 ± 4.41	0.6015
Motor improvement OFF (%)	40.26% ± 41.92%	39.84% ± 42.43%	0.9486

Continuous data with normal distribution are presented as mean ± standard deviation. The other data are presented as median (25th and 75th percentiles). ^a^ Mann–Whitney U tests. ^b^ Chi-square test. Unindicated comparisons were conducted using independent *t*-tests. Significant *p*-values are marked in bold. LEDD: levodopa equivalent daily dose; uPVP: unilateral pneumocephalus volume percent; MDS-UPDRS: Movement Disorder Society Unified Parkinson’s Disease Rating Scale; ON: on-medication condition; OFF: off-medication condition.

**Table 2:** Results of generalized linear model analyses for factors related to the Euclidean distance of the active contact displacement.

	Beta (β)	Standardized Beta (β)	VIF	Corrected *p*-Value
ED of active contact displacement				
Sex	−0.1419	0.0630	1.048	**0.0384**
uPVP	15.1220	4.7305	1.021	**0.0050**
Brain volume percent	0.8099	0.5875	1.046	0.1698

Significant *p*-values are marked in bold. *p*-values were adjusted by FDR correction. VIF: variance inflation factor; ED: Euclidean distance; uPVP: unilateral pneumocephalus volume percent.

**Table 3:** Results of generalized linear model analyses for factors related to the efficacy of subthalamic nucleus deep brain stimulation on motor symptoms.

	Beta (β)	Standardized Beta (β)	VIF	Corrected *p*-Value
Motor improvement ON				
Sex	−1.9999	0.6215	1.067	**0.0031**
Levodopa response	−11.5784	1.5193	1.026	**<0.0001**
Brain volume percent	12.6052	5.7884	1.064	**0.0313**
ED of active contact displacement	−1.5757	0.7256	1.035	**0.0313**
Motor improvement OFF (%)				
Sex	−0.1048	0.0665	1.067	0.1759
Levodopa response	−0.2461	0.1626	1.026	0.1759
Brain volume percent	0.7364	0.6194	1.064	0.2361
ED of active contact displacement	−0.2196	0.0776	1.035	**0.0209**

Significant *p*-values are marked in bold. *p*-values were adjusted by FDR correction. VIF: variance inflation factor; ED: Euclidean distance; ON: on-medication condition; OFF: off-medication condition.

## Data Availability

The data supporting the findings of this study are available on request from the corresponding author. The data are not publicly available due to privacy or ethical restrictions.

## Supplementary Materials

The following supporting information can be downloaded at https://www.mdpi.com/article/10.3390/jcm12247561/s1: Part A. Inclusion and exclusion criteria [43]; Part B. Image Acquisition; Part C. DBS leads location [16,20,32,33,44]; Part D. Electric fields and VTA estimation [21,45]; Part E. Overlap between the VTA and sensorimotor area of the STN [22]. Figure S1: Inclusion and exclusion criteria of PD patients; Figure S2: Electrode location in a single patient on immediate postoperative CT and follow-up postoperative CT; Table S1: Baseline characteristics and coordinates of electrode location comparison between sexes.
