# Supplementary material for: The Related Factors and Effect of Electrode Displacement on Motor Outcome of Subthalamic Nuclei Deep Brain Stimulation in Parkinson’s Disease
Source: J Clin Med. 2023 Dec 8;12(24):7561. doi: 10.3390/jcm12247561 (PMC10744115; doi:10.3390/jcm12247561)
Supplement: Supplementary file 1 [file jcm-12-07561-s001.zip › jcm-2589973-supplementary.pdf]

Supplementary information

Supplemental methods

Part A. Inclusion and exclusion criteria: The inclusion criteria were (1) diagnosis of Parkinson's disease (PD) based on the UK Brain Bank criteria [1], (2) bilateral subthalamic nucleus deep brain stimulation (STN-DBS) surgery, and (3) complete clinical assessment at pre-operation and 1-month follow-up. The exclusion criteria included patients (1) without 3D T1 magnetic resonance imaging (MRI) or thin-section computer tomography (CT) scans, (2) without follow-up CT, (3) a history of prior thalamotomy, (4) an incomplete scale evaluation or programming parameter record, or (3) DBS lead revision or replacement (Supplementary Fig. S1).

Part B. Image Acquisition: All subjects were scanned on 3.0T MRI scanner (Ingenia, Philips Medical Systems, Best, The Netherlands) with a 32-channel head coil. The MRI protocol consisted of the following sequences: a whole-head 3D sagittal T1-weighted-3D magnetization-prepared rapid acquisition gradient echo sequence (voxel size: $1 \times 1 \times 1$ mm^3, slices: 196) and T2-weighted sequence (voxel size: $0.28 \times 0.28 \times 2.5$ mm^3, slices: 25). Immediate postoperative CT and follow-up CT were acquired on a 64-slice CT scanner (GE medical System, Milwaukee, WI, USA) with a spatial resolution of $0.49 \times 0.49 \times 0.625$ mm^3. Images were acquired in axial order at 120 KV and 249 mA.

Part C. DBS leads location: All DICOM files were converted into NIfTI formats with SPM12 (https://www.fil.ion.ucl.ac.uk/spm/software/spm12/). The postoperative CT was co-registered to preoperative MRI using Advanced Normalization Tools (ANTs; http://stnava.github.io/ANTs/) [2]. Next, a brain shift correction method with coarse mask was applied [3]. DBS electrodes were automatically localized and warped into the Montreal Neurological Institute (MNI) using the PaCER algorithm [4] and manually refined as implemented in Lead-DBS [5]. After automated reconstruction, two researchers checked the accuracy and manual adjustment was conducted for inaccurate electrodes. The coordinates of the electrode contacts in MNI space were then converted to the AC-PC space [6].

Part D. Electric fields and VTA estimation: The electric fields (E-fields) were calculated in native space based on the individual optimized stimulation parameters using a finite element method approach established within the SimBio-FieldTrip pipeline [7]. This was performed by solving the static formulation of Laplace equation on a discretized domain represented by a tetrahedral four-compartment model of electrodes and surrounding tissue. Conductivities of 0.33 and 0.14 S/m were assigned to gray and white matter, respectively. The voltage applied to the active electrode contact was introduced as a boundary condition. The surface of volume mesh served as the anode. Volume of tissue activated (VTA) was a binarized version of the E-field,

which is a commonly applied model. The gradient was thresholded for magnitudes above a commonly used value of 0.2 V/mm to define the shape of the VTA [8].

Part E. Overlap between the VTA and sensorimotor area of the STN: The volume of sensorimotor area of the STN in the DISTAL Minimal atlas [9] was transferred to native space using reverse deformation field in each patient. Then, the volume of the STN motor subregion was binarized and resliced to match the resolution of the VTA (voxel size: $0.14 \times 0.14 \times 0.16$ mm^3). Finally, the number of overlapping voxels was investigated, and the overlapping volume was calculated.

References

1. Hughes AJ, Daniel SE, Kilford L, Lees AJ, Accuracy of clinical diagnosis of idiopathic Parkinson's disease: a clinico-pathological study of 100 cases. *J Neurol Neurosur Ps* 1992, *55* (3), 181-184.
2. Avants BB, Tustison N, Song G, Advanced normalization tools (ANTS). *Insight j* 2009, *2* (365), 1-35.
3. Schönecker T, Kupsch A, Kühn A, Schneider G-H, Hoffmann K-T, Automated optimization of subcortical cerebral MR imaging− atlas coregistration for improved postoperative electrode localization in deep brain stimulation. *American journal of neuroradiology* 2009, *30* (10), 1914-1921.

4. Husch A, Petersen MV, Gemmar P, Goncalves J, Hertel F, PaCER-A fully automated method for electrode trajectory and contact reconstruction in deep brain stimulation. *NeuroImage: Clinical* 2018, *17*, 80-89.

5. Horn A, Li N, Dembek TA, Kappel A, Boulay C, Ewert S, Tietze A, Husch A, Perera T, Neumann W-J, Lead-DBS v2: Towards a comprehensive pipeline for deep brain stimulation imaging. *Neuroimage* 2019, *184*, 293-316.

6. Horn A, Kühn AA, Merkl A, Shih L, Alterman R, Fox M, Probabilistic conversion of neurosurgical DBS electrode coordinates into MNI space. *Neuroimage* 2017, *150*, 395-404.

7. Horn A, Reich M, Vorwerk J, Li N, Wenzel G, Fang Q, Schmitz-Hübsch T, Nickl R, Kupsch A, Volkmann J, Connectivity predicts deep brain stimulation outcome in P arkinson disease. *Annals of neurology* 2017, *82* (1), 67-78.

8. Åström M, Diczfalusy E, Martens H, Wårdell K, Relationship between neural activation and electric field distribution during deep brain stimulation. *IEEE Transactions on Biomedical Engineering* 2014, *62* (2), 664-672.

9. Ewert S, Plettig P, Li N, Chakravarty MM, Collins DL, Herrington TM, Kühn AA, Horn A, Toward defining deep brain stimulation targets in MNI space: a subcortical atlas based on multimodal MRI, histology and structural connectivity. *Neuroimage* 2018, *170*, 271-282.

Figures and Tables

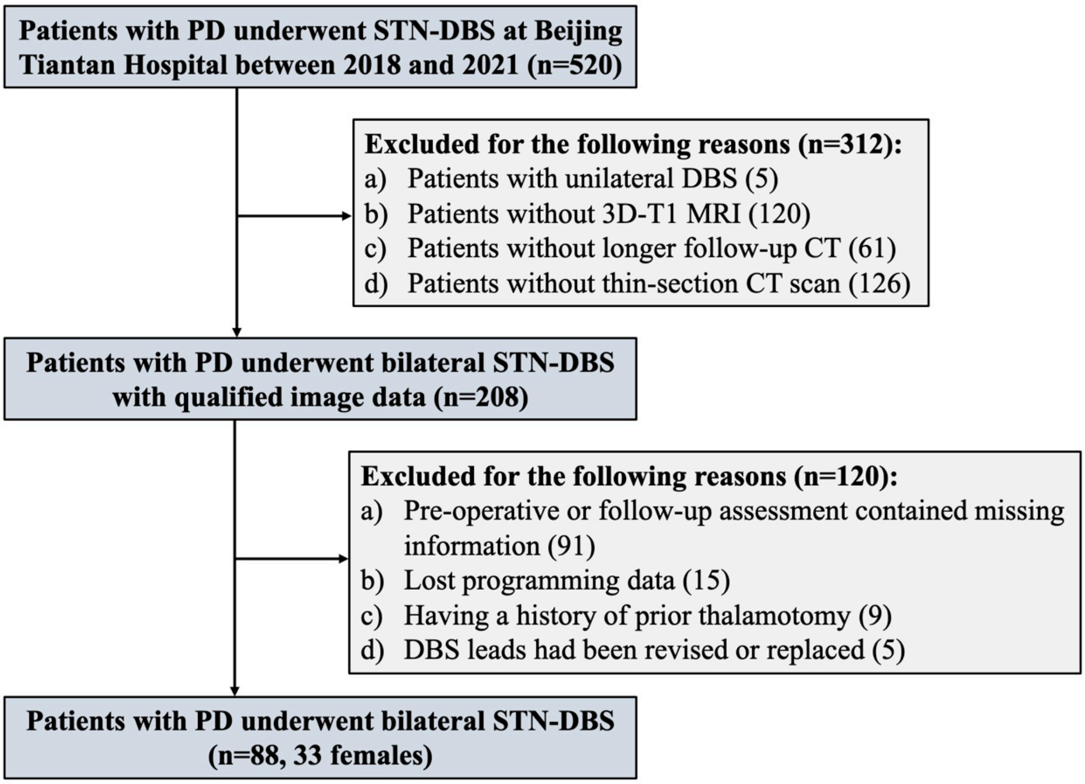

Supplementary Figure S1. Inclusion and exclusion criteria of PD patients. PD: Parkinson's disease; STN-DBS: subthalamic nucleus deep brain stimulation; MRI: magnetic resonance imaging; CT: computer tomography.

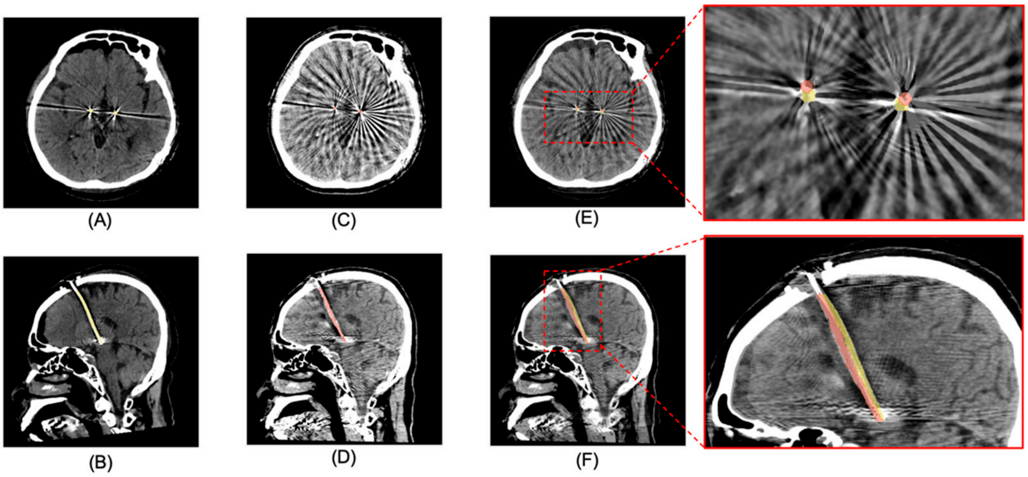

Supplementary Figure S2. Electrode location in a single patient on immediate postoperative CT and follow-up postoperative CT. **A-B.** The electrode location (highlighted in yellow) on immediate postoperative CT in axial view and sagittal view. **C-D:** The position of electrode (highlighted in red) on follow-up CT in axial view and sagittal view. **E-F:** The immediate postoperative CT and follow-up postoperative CT were fused using 3D slicer software to visualize the electrode displacement in axial view and sagittal view. CT: computer tomography.

Supplementary Table S1

Baseline characteristics and coordinates of electrode location comparison between sexes.

	**Males**	**Females**	***P*-value**
Age (year)	61.87 ± 6.03	64.15 ± 6.60	0.1053
Duration of disease (year)[a]	8.00 (6.00, 11.50)	11.00 (5.00,12.00)	0.3476
Hoehn-Yahr stage[a]	3.00 (3.00, 3.00)	3.00 (3.00, 3.00)	0.8339
LEDD (mg/d)[a]	711.50 (600.00, 1000.00)	750.00 (545.50, 925.00)	0.8953
Intracranial volume (mm^3)	1643760.65 ± 124890.51	1396606.68 ± 116166.03	**< 0.0001**
Brain volume percent (%)[a]	72.30% (69.83%, 74.13%)	73.75% (71.56%, 76.25%)	**0.0213**
uPVP (%)[a]	0.33% (0.06%, 0.71%)	0.41% (0.04%, 1.19%)	0.2485
MDS-UPDRS III ON[a]	6.00 (4.00, 8.00)	6.00 (3.25, 9.75)	0.9682
MDS-UPDRS III OFF[a]	14.00 (10.00, 19.00)	16.50 (12.00, 21.00)	0.0942
Levodopa response (%)	56.58% ± 18.78%	61.08% ± 20.11%	0.1384

Continuous data with normal distribution are presented as mean ± standard deviation. The other data are presented as median (25th and 75th percentiles). [a]Mann-Whitney *U* tests. Unindicated comparisons were conducted using independent *t*-tests. Significant comparisons are marked in bold. LEDD: levodopa equivalent daily dose; uPVP: unilateral pneumocephalus volume percent; MDS-UPDRS: Movement Disorder Society Unified Parkinson's Disease Rating Scale; ON: on-medication condition; OFF: off-medication condition.
